# Supplementary material for: Patterns of Intron Gain and Loss in Fungi
Source: PLoS Biol. 2004 Nov 30;2(12):e422. doi: 10.1371/journal.pbio.0020422 (PMC532390; doi:10.1371/journal.pbio.0020422)
Supplement: Table S1 — Also available at http://genes.mit.edu/NielsenEtAl/. (4.3 MB ZIP). [file pbio.0020422.st001.zip › NielsenEtAl/html/1333.html]

AN4691.1.NCU02128.1.MG02544.1.FG08511.1


```
 CLUSTAL W (1.82) Multiple Sequence Alignments - Introns Inserted


Sequence 1: NCU02128.1	371 aa
Sequence 2: FG08511.1	345 aa
Sequence 3: MG02544.1	353 aa
Sequence 4: AN4691.1	358 aa
Alignment Length: 385 aa
Number Identitical Residues: 165 aa
Alignment Score (without introns) 7761


MG02544.1 	MLGTKLTRAASRLLLPSARPIASTTLTATTTTAAARTISSSASNLRPDTKDKD-LPKTA-
NCU02128.1	---MATRAVPRALRASNIFSTAARPTIQTILPSSQRAFHISAIRLQKKKKNTTTTTPGA-
FG08511.1 	------MSFAPSLRLC-VRRVAASPAIR-------PCFTFTAAR---KLHNVPPRQDKP-
AN4691.1  	----MFRPAILRQVVRAVDQSTRPAALVAGPHQFIRPLSTTVSRAQRDDKNDPLTKATTK
          	                     :  .   :        :  :. .   . ::       ..

MG02544.1 	----------GSYARTDASVQVEYPEA-HELPSSEPVKG-AGRAGINVKPTLATFSLQGK
NCU02128.1	----------GAFARTDDSITVEYPEDPSKLPSSQPVDSGIGRAGQNVFPTLATFSLQGK
FG08511.1 	----------GKYAQTDPQIEVEYPED-HELPSSEPVS---GAGGQYVKPTLPTFTLDGH
AN4691.1  	APGGAAGEHEGKYARTDENVVIEYPDD-ADMPRSPVVQ---GRGGMHFKRTLAQFSLENR
          	:...::.. .* :*:** .: :***:   .:* *  *.   * .*  .  **. *:*:.:

MG02544.1 	VGVVTGGARGLGLVM~GQGMVVSGADLAIVDLNL~DEAREQANAIVEQ2L---------~
NCU02128.1	VGVVTGGARGLGLVM~GQGIVVSGGDLAIVDLNK1EEATKQAQNIVET~FKKDYPSAKK2
FG08511.1 	VGIVTGGARGLGLVM~GQGMVFSGSNLALVDMNK1EEAEKQTSLIIEE~FKKENPRARR2
AN4691.1  	VTLVTGGAR-----V1SPEVTVANS------IPG~DEAEESARKLVEQ~FQKENPGLEQ2
          	* :******     : .  :..:..      :   :** :.:  ::*  :... .     

MG02544.1 	-PKVTAHYADVSSEESVNACIEDIVAQHGKIDSLVTSAGFTENFEATQYPIDRMRKLFGV
NCU02128.1	IPKVTAHYADVSDPASVDACIAEIVAEHGKIDNLVTSAGFTENFEAVNYPVDRLRKLWGV
FG08511.1 	IPKVTAHYADVSDPESVEACVAEVVKEHGKIDNLVTSAGFTENFEAVNYPIDRLRKLWAV
AN4691.1  	LPNVTAHYADVSNPDSVNNALSEVIAKHGKIDHLVTSAGFTENFDAISYPYDRMQKLWGV
          	 *:*********.  **: .: ::: :***** ***********:* .** **::**:.*

MG02544.1 	NFDGTYLFATSVAKHL~MARQAAGSMVFIGSMSGAIVNVPQPQAPYNSVKAAVRHLAASL
NCU02128.1	NVDGTYLFATAIARHL0MDRNSPGSLVFIGSMSGSIVNVPQPQTPYNASKAAVRHLAASF
FG08511.1 	NVDGTYLFATSVARHL~MQRKAPGSIVMIGSMSGSIVNVPQPQAPYNAAKAGVRHLAASL
AN4691.1  	NVDGTYLFATGVAKHL~MERKAPGSIVMIGSMSGAIVNVPQPQAPYNAAKAAVRHLASSF
          	*.********.:*:** * *::.**:*:******:********:***: **.*****:*:

MG02544.1 	AVEWAHAGIRVNCISPGYMLTAL~TKKILDDKPDLKKKWTSLIPQGRMGAPEDLMGPVTF
NCU02128.1	AVEWAKAGIRVNCISPGYMLTAL~TKKILDENPDLKEKWTSLIPQGQMGNPEDLMGPVTF
FG08511.1 	AVEWAQANIRVNCISPGYMLTAL2TQKILDDNPDLKAKWTSLIPQGKMGQPQDLMGPVAF
AN4691.1  	AVEWAGHGIRVNCISPGYMLTAL2TRKILDENPELRDKWISLIPTGKMGVPEDLMGAVTF
          	*****  .*************** *:****::*:*: ** **** *:** *:****.*:*

MG02544.1 	LLSD~ASVYVTGADIRVDGGYTVI~----~----
NCU02128.1	LLSD~ASRYVTGADLRVDGGYTCT2YLGM0FYCG
FG08511.1 	LLSD0ASSYVTGADIRVDGGYTVT~----~----
AN4691.1  	LLSD~ASKYITGADLRVDGGYTLT~----~----
          	**** ** *:****:*******
```
